# Ginsenoside Prolongs the Lifespan of *C. elegans* via Lipid Metabolism and Activating the Stress Response Signaling Pathway

**DOI:** 10.3390/ijms22189668

**Published:** 2021-09-07

**Authors:** Xiaoxuan Yu, Hui Li, Dongfa Lin, Weizhuo Guo, Zhihao Xu, Liping Wang, Shuwen Guan

**Affiliations:** 1 Key Laboratory for Molecular Enzymology and Engineering, The Ministry of Education, Jilin University, Changchun 130012, China; yuxx18@mails.jlu.edu.cn (X.Y.); wanglp@jlu.edu.cn (L.W.); 2 School of Life Sciences, Jilin University, Changchun 130012, China; lihui18@mails.jlu.edu.cn (H.L.); lindf1317@mails.jlu.edu.cn (D.L.); guowz1318@mails.jlu.edu.cn (W.G.); xuzh1319@mails.jlu.edu.cn (Z.X.); 3 Engineering Laboratory for AIDS Vaccine, Jilin University, Changchun 130012, China

**Keywords:** *Panax ginseng*, lifespan, lipid metabolism, stress-resistant, *Caenorhabditis elegans*

## Abstract

*Panax ginseng* is a valuable traditional Chinese medicine in Northeast China. Ginsenoside, the active component of ginseng, has not been investigated much for its effects on aging and its underlying mechanism(s) of action. Here, we investigated the effects of total ginsenoside (TG), a mixture of the primary active ginsenosides from *Panax ginseng*, on the lifespan of *Caenorhabditis elegans* (*C. elegans*). We found that TG extended the lifespan of *C. elegans* and reduced lipofuscin accumulation. Moreover, TG increased the survival of *C. elegans* in response to heat and oxidative stress via the reduction of ROS. Next, we used RNA-seq to fully define the antiaging mechanism(s) of TG. The KEGG pathway analysis showed that TG can prolong the lifespan and is involved in the longevity regulating pathway. qPCR showed that TG upregulated the expression of *nrh-80*, *daf-12*, *daf-16*, *hsf-1* and their downstream genes. TG also reduced the fat accumulation and promoted lipid metabolism. Moreover, TG failed to extend the lifespan of *daf-16* and *hsf-1* mutants, highlighting their role in the antiaging effects of TG in *C. elegans*. The four main constitution of TG were then confirmed by HPLC and included ginsenoside Re, Rg_1_, Rg_2_ and Rd. Of the ginsenosides, only ginsenoside Rd prolonged the lifespan of *C. elegans* to levels comparable to TG. These findings provided mechanistic insight into the antiaging effects of ginsenoside in *C. elegans*.

## 1. Introduction

Aging is defined as a progressive decline in tissue, organ and neuronal function [1]. With the elucidation of the signaling pathways that influence aging, strategies to prolong the lifespan and improve human health have been explored. Antioxidants are one such example, which delay aging and promote health [2,3]. Antioxidants are commonly associated with stress responses and can reduce reactive oxygen species levels in vivo. In addition, recent figures have suggested that lipid metabolism plays a key role in aging, and specific lipids and lipid-related molecules can regulate the lifespans of a variety of model organisms (e.g., *C. elegans*, *Drosophila melanogaster*, mosquitoes and rodents) [1,4]. 

*Panax ginseng* is an important traditional Chinese medicine in Northeast China that has gained popularity. Ginseng root has been widely used for centuries in Chinese medicine as a panacea that prolongs the lifespan [5]. White and red ginseng are obtained through the processing of fresh ginseng root but have differential functions. Ginseng has been extensively studied and is used in clinics for a range of disease states. Ginseng contains ginsenosides, polysaccharides, peptides and fatty acids [6,7]. The majority of the pharmacological effects of ginseng root can be attributed to ginsenosides, a diverse group of steroidal saponins [7]. The positive effects of ginsenosides have been shown in the CNS and in the cardiovascular, endocrine and immune systems [8,9,10]. Ginsenosides have been ascribed antineoplastic [11], anti-inflammatory [12] and antioxidant activity [13], which have good medicinal and commercial prospects. Ginsenosides can be utilized by *C. elegans* as a sterol substitute and play an important role in the growth and recovery of nematodes in a cholesterol-deprived medium [14,15]. Ginseng extract can resist the negative effects of heat stress [16]. Studies on *C. elegans* have shown the promising efficacy of certain saponins in reversing age-related declines via antioxidant regulation and longevity signaling pathways [17]. Sun Ginseng can effectively inhibit the proliferation and differentiation of adipocytes and can reduce the fat accumulation in *C. elegans*, which also suggests that the active substance in ginseng that plays the function of reducing the fat accumulation may be ginsenosides [18]. Ginseng extract consists of a mixture of the primary active ginsenosides. Although ginseng possesses antiaging effects, the molecular mechanism(s) of how total ginsenoside (TG) delays aging through the lipid metabolism pathway remain undefined. 

In this study, we used a *Caenorhabditis elegans* (*C. elegans*) model to evaluate the antiaging effects of TG and its underlying mechanism(s) of action. High-performance liquid chromatography (HPLC) was also performed to identify the active constituents of TG. We found that TG and ginsenoside Rd treatment significantly extend the lifespan of *C. elegans* through a shared mechanism. A subsequent genetic analysis revealed that TG and ginsenoside Rd extend the lifespan of *C. elegans* through a shared mechanism involving *nhr-80, daf-12, daf-16* and *hsf-1*.

## 2. Results

### 2.1. Effects of TG on the Lifespan of C. elegans

**Figure 1:**
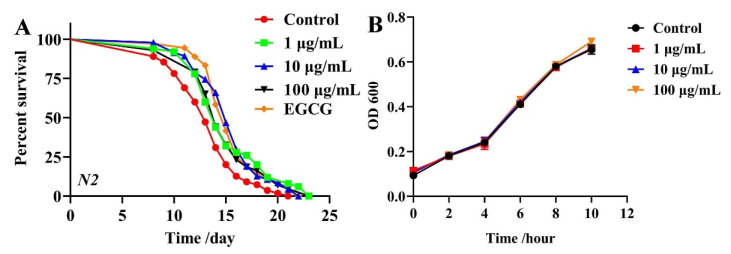
Effects of TG on the lifespan of *C. elegans* and *E coil*. OP50. (**A**) Survival of *C. elegans* treated with 0.1% DMSO (control); 500 μM EGCG (positive control) and 1, 10 and 100 μg/mL TG. TG produced its optimal effects at 10 μg/mL, extending the lifespan of *C. elegans* by up to 9.11 ± 2.04%, *n* = 3 (~80 individuals per group), Kaplan–Meier survival analysis with the log-rank test. (**B**) OP50 in LB medium containing 1, 10 and 100 μg/mL TG. TG did not inhibit the growth of OP50 during any bacterial growth phase. Data were analyzed by a Student’s *t*-test using GraphPad 8. Values are presented as the mean ± SEM.

To investigate the effects of TG on the lifespan of *C. elegans*, we compared to the control group the lifespan curves of the three test concentrations (1 μg/mL, 10 μg/mL and 100 μg/mL and 500 μM EGCG as a positive control), which showed a significant rightward shift (Figure 1A and Table S3), suggesting that TG treatment extended the lifespan of *C. elegans.* Moreover, the most significant effect occurred at 10 μg/mL, which increased the mean lifespan by 9.11 ± 2.04% (Table S3). These data confirmed the ability of TG to prolong the lifespan of *C. elegans*.

Previous studies showed that the metabolism of OP50 influences the lifespan of *C. elegans* and that ginsenosides possess antibacterial effects [19,20,21]. Live OP50 was used in all the experiments. To eliminate the influence of bacterial growth and metabolism on the lifespan of *C. elegans*, we explored whether TG influences the growth and metabolism of OP50. Growth curves of OP50 in LB liquid media containing 1, 10 and 100 μg/mL TG were determined. TG had no significant effects on the proliferation of OP50 (Figure 1B). These results suggested that the TG treatment effectively extended the lifespan of *C. elegans* at 10 μg/mL. This concentration was selected for subsequent experiments.

### 2.2. TG Has No Effects on the Fecundity and Body Size of C. elegans

**Figure 2:**
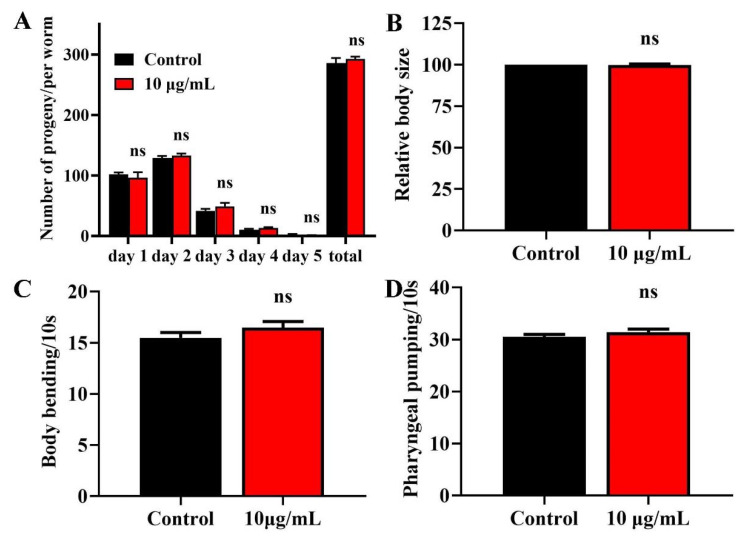
Effects of TG on the breeding population, body size, body bending and pharyngeal pumping in *C. elegans*. (**A**) Breeding of *C. elegans* treated with or without TG. The number of eggs were monitored on day 1 of adulthood until day 5. (**B**) Worms treated with TG showed no significant changes in body size. (**C**) Worms treated with TG showed no significant changes in body bending. (**D**) Worms treated with TG showed no significant changes in pharyngeal pumping.

Lifespan extension is related to a loss of fecundity [22]. We therefore investigated the effects of TG treatment on the reproduction of *C. elegans*. We observed no significant difference in daily egg production and total progeny production following TG treatment (Figure 2A), suggesting that the TG-mediated lifespan extension of *C. elegans* was not dependent on sacrificial reproduction. The pretreatment with TG also caused no changes in the body length (Figure 2B). All of these suggested that TG had no effects on the normal growth and development.

### 2.3. TG Has No Effect on the Body Bending and Food Intake of C. elegans

Muscle cells gradually lose vitality during aging, leading to the loss of locomotion and pharyngeal pumping [23,24]. We measured the body bending rates and pharyngeal pumping of TG treatment as an indicator of muscle change. TG treatment had no effect on body bending or the pharyngeal muscle contractility of *C. elegans* (Figure 2C,D). Pharyngeal pumping also reflects the food intake, which indicated that TG had no effect on the food intake of *C. elegans.* The pretreatment with TG can effectively delay the degradation of muscle performance.

### 2.4. TG Reduces Lipofuscin Accumulation

**Figure 3:**
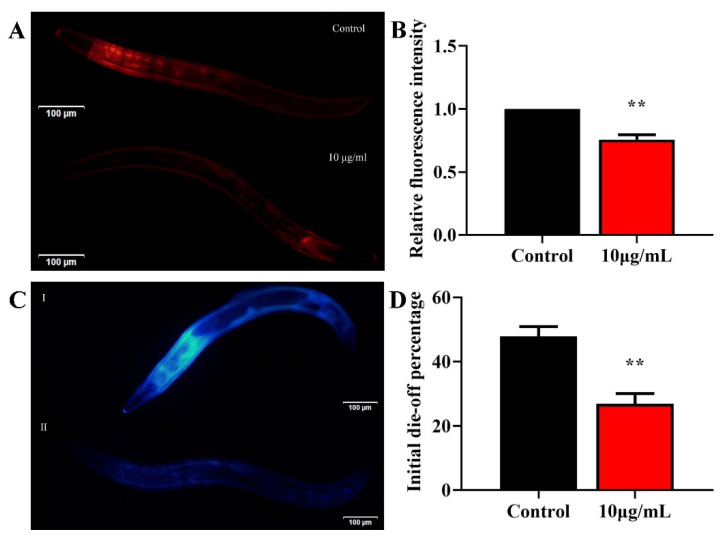
Effects of TG on the lipofuscin accumulation in *C. elegans*. (**A**–**D**) Worms were treated with TG for 8 days at 20 °C. Lipofuscin accumulation was measured using blue (Ex/Em 340/430 nm) and red autofluorescence (Ex/Em 546/600 nm). TG significantly reduced the lipofuscin accumulation in *C. elegans*. The data were analyzed using a Student’s *t*-test. The values were shown as the mean ± SEM, ** *p* < 0.01.

To understand whether TG treatment increases the health span, we evaluated the effects of 10 μg/mL TG on age-associated lipofuscin changes. Lipofuscin is often used as a marker of aging [24,25]. The accumulation of lipofuscin may diminish the autophagocytotic capacity and interfere with the recycling of cellular components [26]. Standardization regarding the excitation/emission wavelengths for the detection of lipofuscin are lacking [25]. It has been reported that blue autofluorescence (excitation/emission centered on 340/430 nm) increases by only low levels before and after death, which may reflect the fraction of dead or almost-dead individuals in the sample. In contrast, red autofluorescence (excitation/emission centered on 546/600 nm) increases linearly over time and correlates with the future lifespan of each individual [25]. We therefore used two excitation wavelengths to detect lipofuscin. TG treatment significantly reduced the lipofuscin deposition compared to the control group (Figure 3A–D). Altogether, TG treatment significantly reduced age-associated lipofuscin accumulation, which can be considered an improvement in the health span of *C. elegans*.

### 2.5. TG Increases Survival and Reduces ROS Levels in Stress-Induced C. elegans

**Figure 4:**
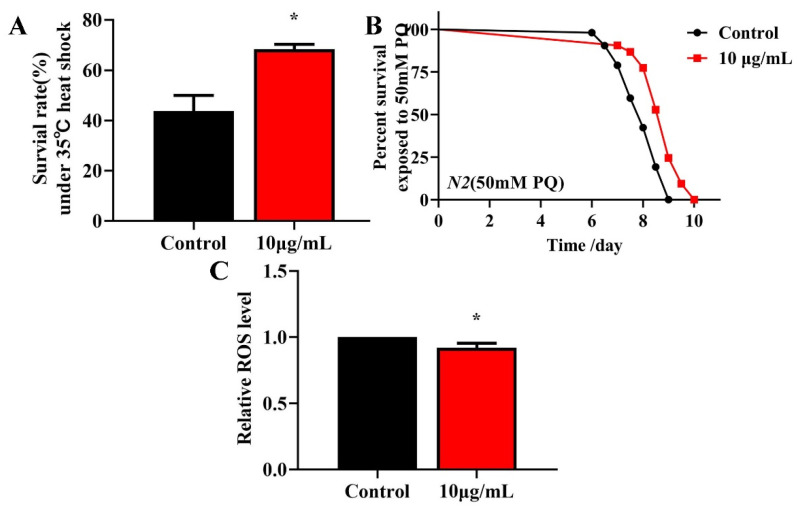
Effects of TG pretreatment on the stress resistance and ROS accumulation in *C. elegans*. (**A**) TG significantly increased the survival of worms under heat stress. (**B**) The worms were treated with or without TG and exposed to 50 mM paraquat. (**C**) Relative levels of ROS in *C. elegans* following the treatment with TG. TG decreased the ROS levels in the worms, evidenced by the quantification of the fluorescence intensity. The data were analyzed using a Student’s t-test. The values were shown as the mean ± SEM, * *p* < 0.05.

Longevity is associated with survival under conditions of oxidative or heat stress [27,28]. To investigate whether TG treatment enhances the stress resistance, L4 worms were pretreated with 10 μg/mL TG for 3 days at 20 °C and exposed to 50 mM paraquat (an intracellular ROS generator) or heat stress (35 °C). The pretreatment with TG significantly increased the survival of worms under stress conditions (Figure 4A,B), indicating that TG increases the resistance of *C. elegans* to oxidative and heat stress. According to the Free Radical Theory of Aging, excessive ROS levels are responsible for the process of aging [29]. Moreover, heat stress leads to ROS accumulation [30,31]. The fluorescent probe H_2_DCFDA can be used to determine the ROS levels in *C. elegans.* The results showed that the TG treatment reduced the intracellular ROS levels under heat stress (Figure 4C). Collectively, the pretreatment with TG not only prolonged the lifespan of worms but also improved their ability to resist stress. Part of the reason for that may be that TG reduced the ROS levels in *C. elegans*.

### 2.6. Genome-Wide Transcriptional Profiling of C. elegans

**Figure 5:**
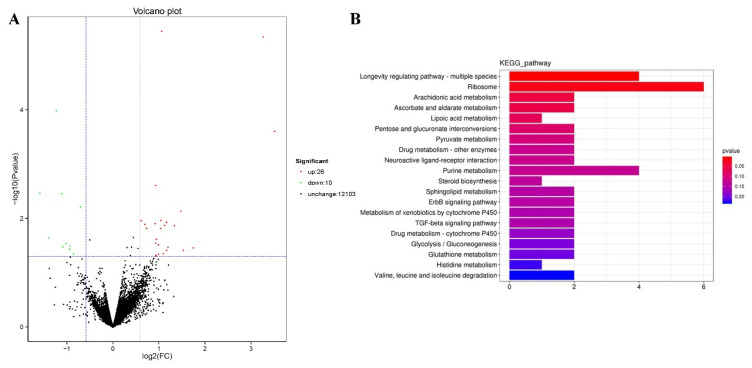
Modulated pathways in TG treated in *C. elegans*. (**A**) A total of 26 genes were upregulated and 10 genes were downregulated following the TG treatment (FDR < 0.05 and fold change > 1.5). (**B**) The KEGG analysis of differentially expressed genes in TG-treated *C. elegans*.

**Figure 6:**
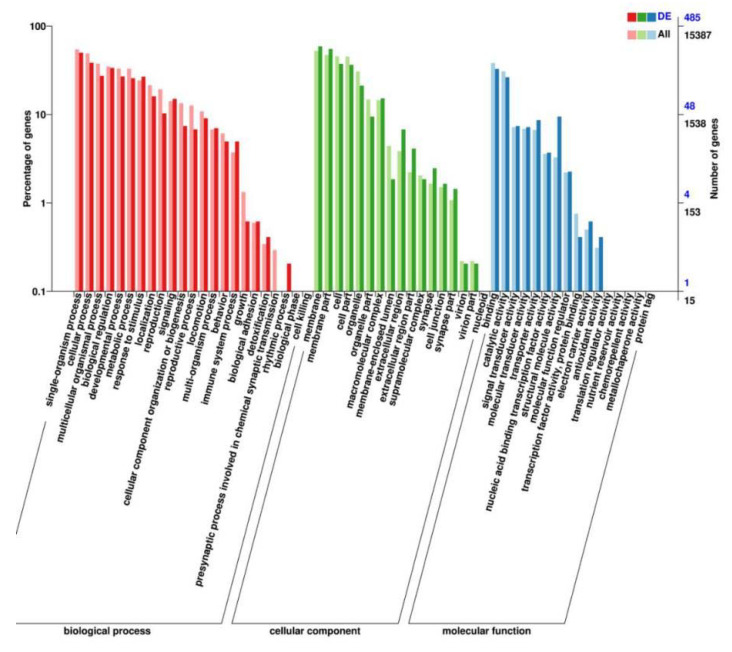
GO analysis of the genes significantly regulated in TG-treated *C. elegans*.

We performed RNA-seq to identify the genes mediating the effects of TG on lifespan extension on a more global scale. Following 3 days of TG treatment, 26 genes were upregulated and 10 were downregulated (fold change > 1.5, *p* < 0.05; Figure 5A). A larger number of GO terms were enriched in upregulated genes, namely single-organism processes, cellular processes, membranes, membrane part binding and catalytic activity (Figure 6).

The functional description of differential genes in the WormBase database revealed that the top five most significantly up and downregulated genes were influenced by the nuclear receptor *daf-12* and downstream of the insulin/IGF-1 signaling pathways *(daf-2)*, including *daf-16* and *hsf-1* (Table 1 and Table 2). Differentially expressed genes were concentrated in the germ cells, intestinal cells and neurons, indicating that the effects of TG on *C. elegans* were tissue-specific.

**Figure 7:**
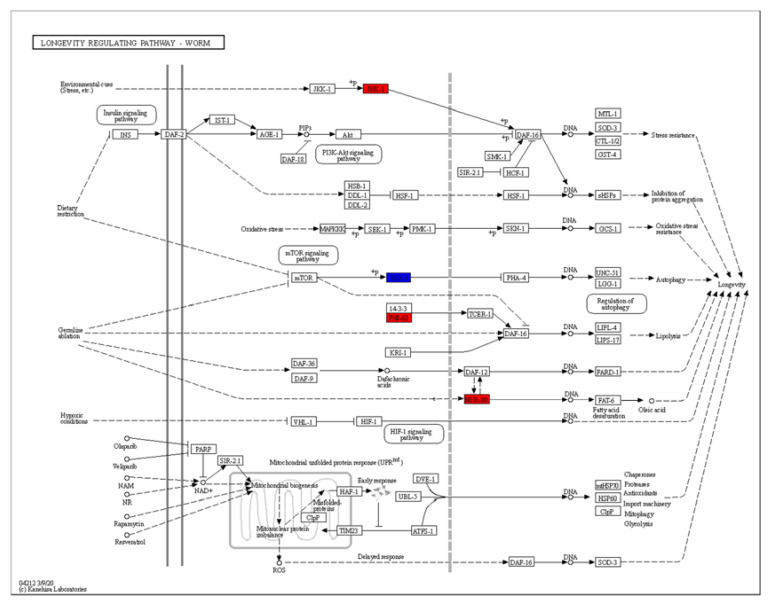
The KEGG analysis of differentially regulated genes.

The KEGG (Kyoto Encyclopedia of Genes and Genomes) analysis revealed dramatic changes in the ribosomes and the metabolism of arachidonic acid, ascorbate, aldarate, lipoic acid and purine (Figure 5B). Differentially expressed genes enriched in the lifespan regulatory pathways showed that *jnk-1*, *phi-62* and *nhr-80* were significantly upregulated (Figure 7). *Jnk-1* and *phi-62* were upstream of the transcription factor DAF-16, which activates DAF-16 and promotes the lifespan of *C. elegans*. As a known activator of DAF-16, JNK-1 is activated in response to cellular stress [32,33]. PHI-62 influences DAF-16-dependent transcription through its interaction with TCER-1 [34]. Moreover, germline ablation and dietary restrictions regulate the stress resistance, lipid metabolism and protein aggregation through DAF-16 and NHR-80 (Figure 7). The nuclear hormone receptor NHR-80 interacts with DAF-12 (Figure 7). According to Table 1 and Table 2, we speculate that TG mediates the longevity of *C. elegans* by influencing the downstream transcription factors of insulin/IGF-1 signaling, DAF-12 and NHR-80. These genes were explored in subsequent experiments.

### 2.7. TG Extends the Lifespan of C. elegans through Lipid Metabolism Signaling Pathway

**Figure 8:**
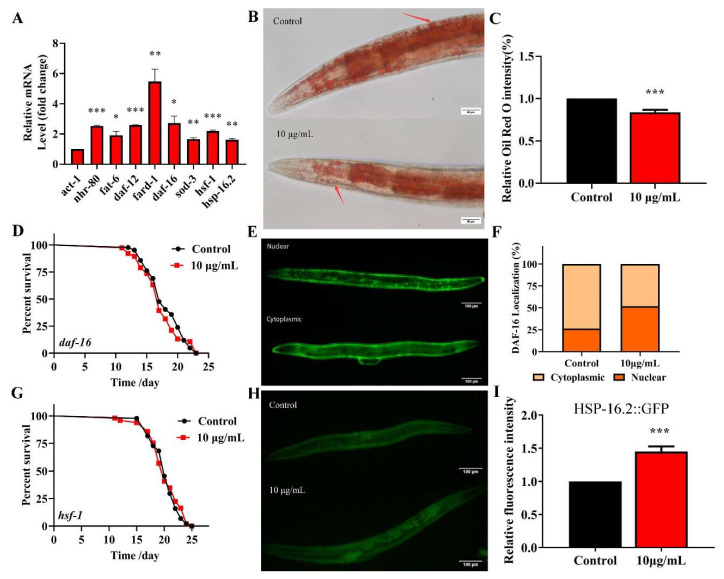
TG reduces the fat accumulation and increases the expression of *nhr-80*, *daf-12*, *daf-16* and *hsf-1* in *C. elegans*. (**A**) TG enhances the expression of *nhr-80*, *fat-6*, *daf-12*, *fard-1*, *daf-16*, *sod-3*, *hsf-1* and *hsp-16.2*. (**B**,**C**) The fat content in *C. elegans* treated with or without TG. The worms were stained with oil red O. TG significantly reduced the fat content in *C. elegans*. (**D**) The mean lifespan of *daf-16 (mgDf50)* treated with or without TG. TG could not extend the lifespan of *daf-16 (mgDf50)*. (**E**,**F**) DAF-16::GFP expression in TG-treated worms. (**G**) The mean lifespan of *hsf-1* *(sy441)* with or without TG. TG failed to extend the lifespan of *hsf-1 (sy441)*. (**H**,**I**). The quantification of HSP-16.2::GFP fluorescence in the presence or absence of TG. TG significantly increases the expression of HSP-16.2 in *C. elegans*. The data were analyzed using a Student’s t-test. The values were shown as the mean ± SEM, *** *p* < 0.001, ** *p* < 0.01, * *p* < 0.05.

Fatty acid desaturase FAT-6 is a key target of NHR-80 and regulates the lipid metabolism in *C. elegans* [35]. The *fard-1* gene of *C. elegans* is predicted to encode a lipid acyl-CoA reductase, regulated by the nuclear receptor DAF-12. These four genes regulate the lipid-metabolism and longevity [36]. TG significantly upregulates the expression of NRH-80 (Figure 7). We used qPCR to analyze the effects of TG on *daf-12* and *nhr-80* and their downstream genes *fard-1* and *fat-6* (Figure 8A). As predicted, TG significantly increased the expression of all genes; amongst which, *fard-1* mRNA increased by more than five-fold. These data confirmed that the regulatory effects of TG on the lipid metabolism are associated with the increased expression of *daf-12*, *nhr-80* and their downstream genes *fard-1* and *fat-6*. We used Oil red staining to observe the lipid accumulation, and the results showed that 10 μg/mL TG significantly reduced the fat accumulation at day 3 (Figure 8B,C). During the aging process, fat gradually accumulates, even leading to obesity-related diseases [37]. This data suggested that TG activated the lipid metabolism signaling pathway and also improved the health span in *C. elegans* through the lipid metabolism signaling pathway.

### 2.8. TG Extends the Lifespan of C. elegans through Activating the Stress Response Signaling Pathway

DAF-16 activates the expression of proteins associated with the resistance to stress responses, which integrates multiple signaling pathways by shuttling from the cytoplasm to the nucleus [38]. To understand the mechanism(s) through which TG extends the lifespan of *C. elegans* through DAF-16/FOXO, lifespan assays were performed in *daf-16(mgDf50)* worms. We found that TG did not extend the lifespan of *daf-16(mgDf50)* worms (Figure 8D and Table S3), suggesting that *daf-16* is required for the TG-mediated lifespan extension of *C. elegans*. A pretreatment with TG induced the nuclear accumulation of DAF-16 (Figure 8E,F) and increased the expression of *daf-16* mRNA (Figure 8A). Cytoprotective genes such as superoxide dismutase (SOD-3) are upregulated by the IIS-dependent transcription factor DAF-16 [39]. A qPCR analysis showed that *sod-3* was significantly upregulated in *C. elegans* exposed to TG (Figure 8A). These results suggested that the antiaging mechanisms of TG were closely related to DAF-16 and its downstream gene *sod-3*. 

Heat shock factor-1 (HSF-1) is a master regulator of stress responses and longevity [40]. TG treatment improves the survival of *C. elegans* under stress conditions (Figure 4A). The most significant differential genes affected by HSF-1 in the RNA-Seq data were, therefore, investigated (Table 1). We utilized worms defective in *hsf-1, hsf-1(sy441),* for the lifespan assays. The findings suggested that TG treatment did not extend the lifespan of *hsf-1(sy441)* worms (Figure 8G and Table S3). The qPCR analysis showed that the expression of *hsf-1* was significantly upregulated (Figure 8A). These results indicated that *hsf-1* was required for the TG-mediated lifespan extension of *C. elegans*. HSF-1 can activate the expression of heat shock proteins (HSPs), which, in turn, promote longevity and prevent protein aggregation [41,42]. The qPCR analysis showed that *hsp-16*.2 is significantly upregulated in *C. elegans* exposed to TG (Figure 8A). Transgenic *C. elegans* expressing HSP-16.2::GFP were then used to examine the effects of TG on HSP-16.2 (Figure 8H,I). The results suggested that TG enhanced the expression of antioxidant stress-related genes, and TG extended the lifespan of *C. elegans* through activating the stress response signaling pathway.

### 2.9. Analysis of the Major Components of TG and Their Antiaging Effects

**Figure 9:**
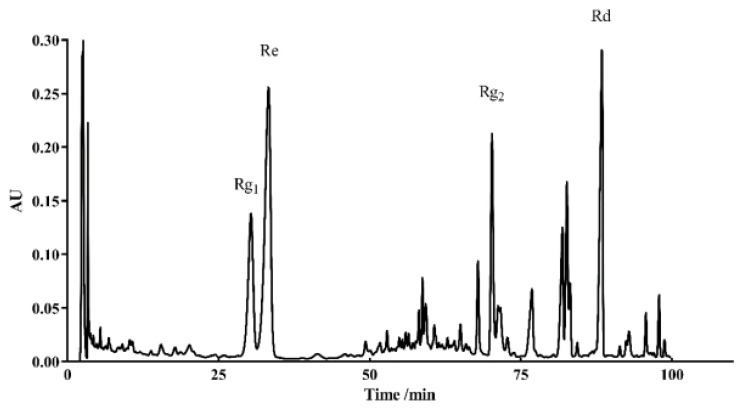
HPLC chromatograms of TG recorded at 203 nm.

**Figure 10:**
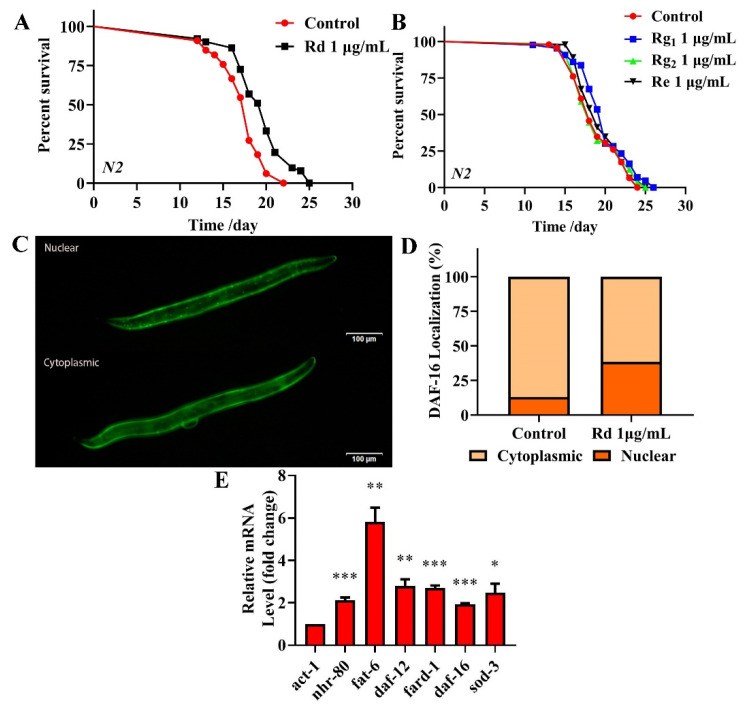
Effects of ginsenoside Rd, Rg_1_, Re and Rg_2_ on the lifespan of *C. elegans*. (**A**) The survival of *C. elegans* treated with 0.1% DMSO (control) and 1 μg/mL Rd, Rg_1_, Re and Rg_2_. Worms exposed to ginsenoside Rd survived longer than untreated worms (*p* < 0.05). (**B**) Ginsenoside Rg_1_, Re and Rg_2_ (1 μg/mL) did not extend the lifespan of *C. elegans*. (**C**,**D**) Fluorescence images of DAF-16::GFP expression in ginsenoside Rd-treated and untreated worms. (**E**) mRNA levels of *nhr-80*, *daf-12* and *daf-16* and the downstream genes *fat-6*, *fard-1* and *sod-3* in *C. elegans* treated with ginsenoside Rd. The data were analyzed using a Student’s t-test. The values were shown as the mean ± SEM, *** *p* < 0.001, ** *p* < 0.01, * *p* < 0.05.

HPLC was used to identify the active components within TG. We observed the presence of ginsenoside Rg_1_, Re, Rg_2_ and Rd, which had relative abundance values of 10.91%, 24.61%, 20.05% and 7.64%, respectively (Figure 9 and Table 3). Rg_1_, Re, Rg_2_ and Rd were then analyzed for their antiaging effects to further investigate the effects of these components on the lifespan of *C. elegans*. Based on the HPLC analysis, the concentrations of the four ginsenosides in TG were approximately 1 μg/mL. The treatment with 1 μg/mL ginsenoside Rd significantly prolonged the mean lifespan of *C. elegans* by 13.26 ± 2.25% (Figure 10A and Table S3). Ginsenosides Rg_1_, Re and Rg_2_ had no significant effect on the mean lifespan (Figure 10B and Table S3). 

To confirm that the antiaging mechanisms of ginsenoside Rd are similar to those of TG, we analyzed the expression of *nhr-80*, *daf-12* and *daf-16* and their downstream genes *fat-6*, *fard-1* and *sod-3* following the ginsenoside Rd treatment. The results showed that ginsenoside Rd significantly increased the expression of *nhr-80*, *daf-12*, *daf-16*, *fat-6*, *fard-1* and *sod-3* (Figure 10E). We next investigated the effects of ginsenoside Rd on DAF-16 using the TJ356 transgenic strain (expressing DAF-16::GFP). We found that the treatment with 1 μg/mL ginsenoside Rd significantly increased the transport of DAF-16 into the nucleus (Figure 10C,D), suggesting a similar mechanism of lifespan extension to that of TG.

## 3. Discussion

Due to the increasing age of the population globally, interest in the development of pharmacological approaches that can delay the process of aging and other aging pathologies has intensified. Recent studies have suggested that ginsenoside can prevent oxidative stress, metabolic disorders and neurodegenerative disease [43,44,45]. Previous studies have suggested that scavenging free radicals could reduce the occurrence of diseases and effectively prolong the lives of organisms [2,3]. We found that TG treatment extends the lifespan of *C. elegans* (Figure 1A). Previous studies have found that ginseng extract extends the lifespan of *C. elegans* through a resistance to oxidative stress (*daf-16*) and heat stress (*hsp-1* and *hsp-16.2*) [16]. Our results were mutually verified with the above reports. The TG-mediated extension of the lifespan of *C. elegans* raised the possibility that TG effectively protects *C. elegans* from stress conditions through the reduction of ROS levels (Figure 4A–C). Lipofuscin is an age-related pigment, and it has been shown that lipofuscin accumulate in vivo with the increase of age in *C. elegans* [25]. Our results found that TG treatment significantly extended the lifespan of *C. elegans* through its ability to reduce intestinal lipofuscin (Figure 3A–D).

*C. elegans* is advantageous for antiaging research due to its complete genome sequence [24]. We investigated the possible mechanisms of TG on the lifespan extension of *C. elegans* through RNA-seq. We speculated that TG activates *daf-12*, *nhr-80* and downstream insulin/IGF-1 signaling pathways *(daf-2)* such as *daf-16* and *hsf-1.* DAF-16 is in the FOXO family of transcription factors and regulates the genes involved in promoting stress resistance, fat metabolism and influencing the lifespan [46]. We used traditional methods to verify the RNA-seq datasets. TG treatment activated DAF-16 and HSF-1 and upregulated antioxidant cellular defense genes (Figure 8A). More importantly, *daf-16* and *hsf-1* were required for TG-mediated lifespan extension. HSF-1 and DAF-16 activate the expression of heat shock proteins, which, in turn, promote longevity [28]. The expression of stress-response proteins such as HSP-16.2 were upregulated following TG treatment (Figure 8H,I). The overexpression of the cytoplasmic stress reporter *hsp-16.2* could extend the lifespan of *C. elegans* [47]. These results showed that the beneficial effects of TG were mediated through its ability to prevent oxidative stress and regulate the lipid metabolism.

Gonadal longevity relies on the transcriptional activity of the steroid nuclear receptor DAF-12 and nuclear receptor NHR-80 [48]. NHR-80 is a nuclear transcription factor involved in the regulation of longevity in lysosomal signaling pathways [49]. Ginsenosides could increase the effects of cholesterol on reproduction, growth and development in *C. elegans* [14]. In this study, given the array of metabolic pathways identified (Figure 7), we speculated that TG mediated its effects on the aging of *C. elegans* through metabolism. Two major metabolic receptors, *nhr-80* and *daf-12*, were significantly upregulated following TG treatment, confirming its ability to promote metabolism. After lipid staining, we further determined that TG can reduce fat accumulation (Figure 8B,C). In conclusion, TG extended the lifespan of *C. elegans* through multiple signaling pathways, including lipid metabolism and activating the stress response signaling pathway. Ginsenosides are bioactive components of *Panax ginseng*. TG as an extract of ginseng has a complex composition. HPLC was used to determine the bioactive compounds present in TG. Four ginsenosides were identified (Rg_1_, Re, Rg_2_ and Rd). Lifespan assays were performed for Rg_1_, Re, Rg_2_ and Rd (Figure 10A,B). Only Rd (1 μg/mL) prolonged the lifespan of *C. elegans* and significantly increased the expression of *nhr-80*, *daf-12*, *daf-16*, *fat-6*, *fard-1* and *sod-3* (Figure 10E). The pathways through which ginsenoside Rd promoted the longevity of *C. elegans* were therefore similar to those of TG.

In this study, we used the genome-wide transcriptional profiling of N2 worms to explore the mechanism of the TG-increased lifespan in *C. elegans*. We found that TG and ginsenoside Rd have similar mechanisms in prolonging the lifespan of *C. elegans*. Yet, further studies on the mechanisms of ginsenoside monomers remain to be conducted. This study provided new insight into further studying the antiaging effect of ginsenoside monomers in the future.

## 4. Materials and Methods

### 4.1. Strains and Chemicals

All strains were obtained from the Caenorhabditis Genetics Center (CGC) and are shown in Table S1. The strains were grown and maintained on NGM plates seeded with *E. coli* OP50 (OP50). Total ginsenoside (TG) was purchased from Shanghai YuanYe Biotechnology and dissolved in H_2_O. Ginsenosides standard, Rd, Rg_1_, Re and Rg_2_ were purchased from Dalian Meilunbio and dissolved in DMSO and diluted to 0.01%. Epigallocatechin gallate (EGCG) was purchased from Aladdin (Shanghai, China). The samples were mixed with OP50 and coated on the surface of NGM overnight prior to use.

### 4.2. Lifespan Assays

Lifespan assays were performed at 20 °C. At least 80 synchronized L4 larvae worms were transferred to NGM plates (60 mm in diameter) containing OP50. The plates consisted of 50 μM 5-fluoro-2-deoxyuridine (FUDR, Aladdin, China) to prevent egg laying [50]. Synchronization was defined as experimental day 0. The worms were transferred to fresh plates with or without TG each day. The worms that did not respond to mechanical stimuli were scored as dead.

### 4.3. Antibacterial Assay

TG was diluted in 96-well microtiter plates with OP50. A total of 100 μL of bacterial suspension was incubated at 37 °C and read in a microplate reader (Infinity 200 Pro microplate reader, Tecan Trading, Switzerland). OD600 values were recorded every 2 h.

### 4.4. Body Length Measurements

Synchronized L4 worms were treated with 10 μg/mL TG for 3 days. The worms were exposed to heat treatment at 45 °C for 2 h prior to body length measurements [51]. The images were captured on an Olympus X71 fluorescence microscope (Olympus Co., Tokyo, Japan). The body lengths were measured from the top of the head to the tip of the tail using segmented line tools on ImageJ 15.2v (Rawak Software Inc., Stuttgart, Germany).

### 4.5. Reproduction Assays

A total of 5 synchronized L4 worms were randomly transferred to fresh NGM plates with or without 10 μg/mL TG. The worms were transferred onto fresh NGM plates every 24 h until reproduction ceased. The eggs were allowed to hatch and were counted at the L2 or L3 stage. The daily and total fecundity of each worm were recorded.

### 4.6. Body Bend Assay

Synchronized L4 worms were treated with 10 μg/mL TG for 3 days. The frequency of the body bending of the worms was counted for 10 s in M9 buffer under a microscope (BK1201, Chongqing, China). The number of sinusoidal curves made during locomotion was scored.

### 4.7. Pharyngeal Pumping Assay

Synchronized L4 worms were treated with 10 μg/mL TG for 8 days. The pharyngeal pump of worms was counted for 10 s under a microscope (BK1201, Chongqing, China).

### 4.8. Lipofuscin Assays

Synchronized L4 worms were treated with 10 μg/mL TG for 8 days. The images were captured using an Olympus X71 fluorescence microscope (Olympus Co., Tokyo, Japan). A total of 30 worms were measured and detected by red (Ex/Em 546/600 nm) or blue fluorescence (Ex/Em 350/460 nm) [52]. The worms were separated into Class I (dead or almost-dead individuals) and II (normal) with respect to the blue autofluorescence. Representative images of the two types of worms were recorded. The number of worms in each category were counted. Red fluorescence was measured on ImageJ, and the background signals were subtracted. The scores were the average age pigment fluorescence intensity levels of three independent trials.

### 4.9. Resistance to Thermal Stress

Thermal stress assays were performed as previously described [53]. NGM plates containing 30 animals per plate were treated with 10 μg/mL TG. Survival was measured after 4 h at 35 °C, followed by a recovery period of 12 h at 20 °C. The worms that did not respond to mechanical stimuli were considered dead.

### 4.10. Oxidative Stress Assays

Paraquat-induced oxidative stress assays were performed by incubating the worms with 10 μg/mL TG for 24 h [54]. The worms were then transferred to fresh NGM plates containing 50 mM paraquat (Aladdin, Shanghai, China) and 10 μg/mL TG. Worm survival was monitored every 12 h by touch-provoking movement. Worms that failed to respond to mechanical stimuli were considered dead. Log-rank tests were used for all statistical analyses.

### 4.11. ROS Assessments under Thermal Stress

The ROS levels were quantified using 2′,7′-dichlorofluorescein diacetate (H_2_DCFDA) (Meilunbio, Dalian, China, Ex/Em 470/550 nm) [55]. After exposure to 10 μg/mL TG for 3 days, the worms were washed with cold M9 buffer and stained with 100 μM H_2_DCFDA at 37 ℃ for 30 min. The images were taken on an Olympus X71 microscope (Olympus Co., Tokyo, Japan). ImageJ was used to analyze the gray values. Background signals were subtracted.

### 4.12. Oil Red O Staining

Oil Red O (Aladdin, Shanghai, China) staining was performed as previously described with minor modifications [56]. Following the 10 μg/mL TG treatment, the worms were harvested by washing with cold M9 buffer. Oil red O solution (1%) in isopropanol (Aladdin, Shanghai, China) was diluted in 2% Triton X-100. The worms were fixed for 20 min, stained, washed with M9 buffer and placed on 2% agarose gel pads. ImageJ was used to quantify the mean intensity of Oil red O staining [52]. Background signals were subtracted.

### 4.13. Fluorescence Intensity Quantification Assays

To investigate the fluorescence intensity, transgenic worms (TJ375) were treated with 10 μg/mL TG for 3 days, anesthetized in 10 mM levamisole (Aladdin, Shanghai, China) and placed on a 2% agarose pad. The images were captured using an Olympus X71 microscope and quantified using ImageJ [52].

### 4.14. DAF-16 Nuclear Localization Assays

For the quantification of DAF-16::GFP localization, synchronized L4 larvae TJ356 worms expressing DAF-16::GFP were soaked in M9 buffer containing 10 μg/mL TG for 3 h and analyzed on a fluorescence microscope. The worms were grouped into two categories (cytoplasmic and nuclear) dependent on the localization of DAF-16::GFP. Representative images of transgenic TJ356 worms with cytosolic and nuclear DAF-16::GFP were taken, and the number of worms in each category was counted.

### 4.15. mRNA Extraction and Quantitative Real-Time PCR

The worms were treated with 10 μg/mL TG for 3 days, and the total RNA was extracted from ~2000 worms per experimental condition using the Trizol reagent (TransGen Biotech, Beijing, China). First-stranded cDNA was prepared using commercial qPCR kits (Bimake, Houston, TX, USA). qPCR was performed using the Prism 7500 Real-Time PCR System (Applied Biosystems, Foster City, CA, USA) with SYBR^®^ PCR kits (Bimake, Houston, TX, USA). qPCR consisted of 0.5-μM primers and 1 μL cDNA in a reaction volume of 20 μL. Relative fold changes in the transcript levels were calculated using the 2^−ΔΔ*Ct*^ method. The expression of *act-1* was used as an endogenous control to normalize the total mRNA levels. The primers are shown in Table S2.

### 4.16. RNA Sequencing

Wild-type *C. elegans* treated with 10 μg/mL TG at day 3 were assessed by the Biomarker Technology Company (Beijing, China). The WBcel235 genome was used as a reference for all the analyses. The gene expression was quantified as fragments per Kb of the transcript per million fragments mapped. The formula was as follows:

(1) $$\mathrm{FPKM}=\frac{cDNAFragments}{MappedFragments\left(Millions\right)*TranscriptLength\left(kb\right)}$$

A differential expression analysis of the two samples was performed using edgeR. FDR < 0.05 and fold change > 1.5 were set as the thresholds for significant differential expression.

See the Supplementary Materials for more information.

### 4.17. HPLC Analysis of TG

The ginsenosides standards were obtained from Extrasynthese (Meilun, Dalian, China). The samples were dissolved in methanol and analyzed by HPLC (Waters Delta 600 system, Milford, MA, USA) using a ZORBAX C18 5 μm 4.6 × 250 mm column (Agilent, Santa Clara, CA, USA). The water mobile phase was 0.1% phosphoric acid in water (A), and the organic phase was acetonitrile (B). An isocratic elution of 19% B lasted from 0 to 35 min; at 35–55 min, from 19% B to 29% B; 55–70 min, 29% B and 29–40% solvent B from 70 to 100 min; finally, the column returned to its starting condition at 10 min. The column flow was 1 mL/min, the temperature was kept at 25 °C and the absorbance at 203 nm was monitored.

### 4.18. Statistical Analysis

All experiments were performed in triplicate. The lifespans were compared using GraphPad 8 software (GraphPad Software Inc., San Diego, CA, USA). The *p*-values were calculated using the log-rank test. The numerical data were analyzed using a Student’s *t*-test. The values were shown as the mean ± SEM. The statistical differences were considered significant at *p* < 0.05 (* *p* < 0.05, ** *p* < 0.01 and *** *p* < 0.001).

## 5. Conclusions

**Figure 11:**
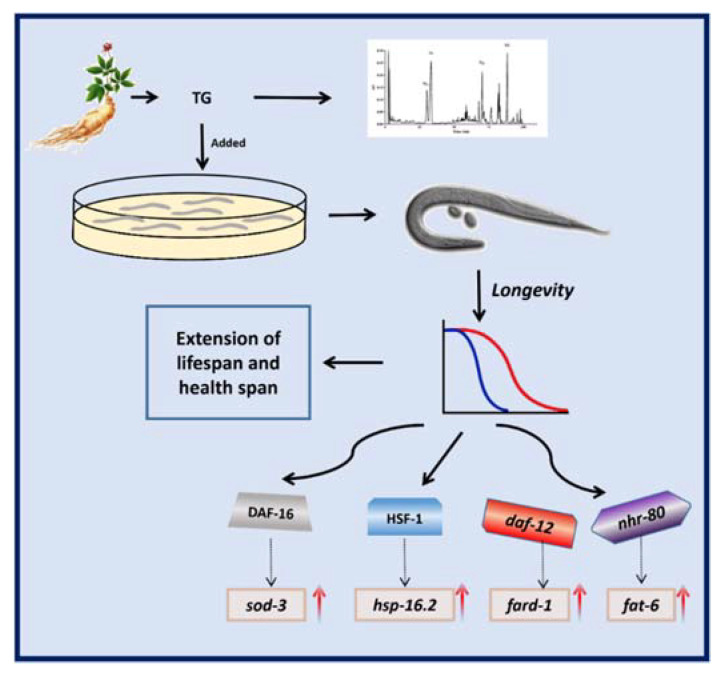
Schematic representation of the mechanism of the TG-extended lifespan.

The results of the present study showed that TG increased the lifespan in *C. elegans* (Figure 11). In summary, we showed that TG promoted stress resistance and the lifespan of *C. elegans*. Moreover, a pretreatment with TG and ginsenoside Rd prolonged the longevity through the lipid metabolism and activated the stress response signaling pathway in *C. elegans*. Further studies are now required to investigate the beneficial effects of ginsenosides on the lifespan and as a potential treatment for age-related diseases.

## Figures and Tables

**Table 1:** Top 5 upregulated genes in response to TG.

	Gene Name	Gene Description (Data from WormBase)
1	*C56C10.7*	Is affected by several genes, including *daf-12*, *sir-2.1* and *pgl-1*. Is predicted to encode a protein with the Protein of unknown function (DUF974) and Trafficking protein particle complex subunit 13. Is an ortholog of human TRAPPC13 (trafficking protein particle complex 13).
2	*F36H5.14*	Is affected by several genes, including *dpy-10*, *hsf-1* and *elt-2*. Is predicted to encode a protein with the MATH domain, MATH/TRAF domain and TRAF-like.
3	*gnrr-1*	Is predicted to have G protein-coupled receptor activity and peptide-binding activity. Human orthologs of this gene are implicated in hypogonadotropic hypogonadism 7 with or without anosmia. Is an ortholog of human GNRHR (gonadotropin releasing hormone receptor).
4	*nspc-2*	Is affected by several genes, including *daf-16*, *prg-1* and *egl-9*.
5	*coa-4*	Is affected by several genes, including *daf-2*, *let-60* and *hsf-1*. Is affected by Cry5B based on microarray studies.

**Table 2:** Top 5 genes downregulated in response to TG.

	Gene Name	Gene Description (Data from WormBase)
1	*F35E8.13*	Is affected by several genes, including *daf-16*, *eat-2* and *sek-1*. Is predicted to encode a protein with ShK domain-like and ShKT domain.
2	*atx-3*	Exhibits thiol-dependent ubiquitin-specific protease activity. Is involved in chemical synaptic transmission. Human orthologs of this gene are implicated in Machado-Joseph disease. Is an ortholog of human ATXN3.
3	*wdr-23*	Exhibits transcription factor-binding activity. *wdr-23* activity is required for the regulation of stress resistance, longevity, and normal growth and development. Is an ortholog of human DCAF11.
4	*Y102A11A.7*	Is affected by several genes, including *daf-16, daf-2* and *skn-1*.
5	*cat-2*	Exhibits tyrosine 3-monooxygenase activity. Is involved in the cellular response to amphetamine, the dopamine biosynthetic process from tyrosine and male mating behavior. Is an ortholog of human TH (tyrosine hydroxylase).

**Table 3:** Contents of the ginsenosides in TG.

Compounds	Retention Time (min)	Peak Area (μV/s)	%Area
Rg_1_	30.350	7,132,106	10.91
Re	33.248	16,092,204	24.61
Rg_2_	67.902	13,108,055	20.05
Rd	70.222	4,992,917	7.64

## Supplementary Materials

The following are available online at https://www.mdpi.com/article/10.3390/ijms22189668/s1, Table S1. All the strains used in this study. Table S2. A list of the primers used for the quantitative real-time reverse transcription-polymerase chain reaction. Table S3. The lifespans of N2 and mutant *C. elegans*.
